# Anti-inflammatory effects of escin are correlated with the glucocorticoid receptor/NF-κB signaling pathway, but not the COX/PGF2α signaling pathway

**DOI:** 10.3892/etm.2013.1128

**Published:** 2013-05-22

**Authors:** HONGSHENG WANG, LEIMING ZHANG, NA JIANG, ZHENHUA WANG, YATING CHONG, FENGHUA FU

**Affiliations:** Department of Pharmacology, School of Pharmacy, Yantai University, Yantai, Shandong 264005, P.R. China

**Keywords:** escin, inflammation, prostaglandin-F2α, nuclear factor-κB, glucocorticoid

## Abstract

In China, escin has been widely used in the clinic as a potent anti-inflammatory drug. Previous studies have indicated that escin exerts its anti-inflammatory effect by enhancing the release of glucocorticoids (GCs) and prostaglandin-F2α (PGF2α), and this has been documented in the drug description. However, our previous studies demonstrated that escin did not increase the secretion of GCs, but instead elevated the protein expression of the GC receptor (GR), which may have repressed nuclear factor (NF)-κB-mediated gene expression. The aim of this study was to determine the functions of NF-κB and PGF2α with regard to the anti-inflammatory effect of escin. We investigated the anti-inflammatory effects of dexamethasone, diclofenac and escin against carrageenan-induced paw edema in rats, and observed that escin exerted a GC-like anti-inflammatory effect. In addition, we studied the role of PGF2α in the anti-inflammatory effect exerted by escin in an acetic acid-induced capillary permeability model in mice. The results revealed that the coadministration of escin and diclofenac, a potent prostaglandin-synthesis inhibitor, did not affect the anti-inflammatory effect of escin. Furthermore, we investigated the function of NF-κB with regard to the anti-inflammatory effect exerted by escin in lipopolysaccharide (LPS)-treated mice, and demonstrated that escin significantly inhibited the expression of NF-κB. These results suggest that escin has a GC-like anti-inflammatory effect, and that its mechanisms may be correlated with the GC receptor/NF-κB signaling pathway, but not the COX/PGF2α signaling pathway.

## Introduction

Escin is the predominant active constituent of *Aesculus hippocastanum* seed extract, which is a triterpene saponin mixture consisting of A, B, C and D escin. Accumulating experimental results in previous studies have suggested that escin exerts potent anti-inflammatory and anti-edematous effects (1–4); consequently, escin is, at present, widely used in the clinic.

The anti-inflammatory mechanisms of escin have been documented in the description of the drug as being due to an increase in the secretion of glucocorticoids (GCs) and prostaglandin-F2α (PGF2α). However, our previous results demonstrated that the anti-inflammatory effect of escin was not dependent on the release of GCs, and, moreover, no immunosuppressive effects were observed with GCs (5). We further identified that the anti-inflammatory effect was correlated with an elevation in the expression level of GC receptor (GR) protein (4).

GCs exert anti-inflammatory effects by binding to GRs, which, upon activation, translocate to the nucleus and inhibit proinflammatory transcription factors, such as nuclear factor-κB (NF-κB) (6). It has been reported that escin may aid tumor suppression by downregulating NF-κB (7,8). However, whether the anti-inflammatory effects of escin are mediated by the GR/NF-κB signaling pathway has remained undetermined.

PGF2α is a member of the prostaglandin (PG) family that is important in the modulation of inflammation. PGF2α differs from other PGs, such as PGF1 and PGE2, in that it is able to contract blood vessels, facilitate venous return and improve lymph transport, and thus counteracts the exudation and edema induced by inflammatory mediators (9–13). It has been indicated that escin is able to increase the secretion of PGF2α from vascular endothelial cells (14–16); however, it has not been determined whether the anti-inflammatory mechanisms of escin occur as a result of this increased secretion of PGF2α.

In the present study, we investigated the GC-like anti-inflammatory effects of escin, and studied the functions of PGF2α and NF-κB in these effects.

## Materials and methods

### Animals

Male Swiss mice (weight, 18–22 g) and male Wistar rats (weight, 160–200 g) were purchased from the Experimental Animal Center of Shandong Luye Pharmaceutical Co., Ltd. (Yantai, China). All experimental procedures performed in this study were executed in accordance with the Yantai University (Yantai, China) guidelines for the care and use of laboratory animals, and were approved by the ethics committee of the university. The animals were housed in diurnal lighting conditions (12/12 h) and were allowed free access to food and water.

### Drugs and materials

The escin used in the experiments was in the form of sodium aescinate, obtained as a lyophilized powder in a 5 mg vial (batch no. 201004144; Shandong Luye Pharmaceutical Co., Ltd.). Diclofenac sodium tablets were purchased from the Sichuan Shuzhong Pharmaceutical Group (batch no. 0908032; Guanghan, China), and diclofenac sodium injections were obtained from the Chongqing Laimei Pharmaceutical Co., Ltd. (batch no. 20100805; Chongqing, China). The dexamethasone sodium phosphate injections used in the study were purchased from the Tianjin Pharmaceutical Group Corp. (batch no. 1009231; Hedong, China), while the λ-carrageenan was purchased from Sigma-Aldrich (batch no. 1408463V; Shanghai, China). The *Escherichia coli* O55:B5 lipopolysaccharide (LPS) was purchased from Sigma-Aldrich. The primary and secondary antibodies to NF-κB were obtained from the Beyotime Institute of Biotechnology (Haimen, China).

### Carrageenan-induced paw edema model

Forty Wistar rats were randomly divided into control, dexamethasone (4 mg/kg), escin (1.8 mg/kg) and diclofenac (6 mg/kg) groups. Carrageenan (0.1 ml, 1% w/v) was injected into the right-hind paw (sub-plantar) of the rats, and the corresponding drugs were administered (intragastrically for diclofenac, and intravenously for dexamethasone and escin) 30 min later. The paw volume was measured with a hydroplethysmometer (Shandong Academy of Medical Sciences, Jinan, China) prior to the irritant injection, and at selected intervals (1, 2, 4, 6, 8, 12, 18 and 24 h) subsequent to the administration of the drugs. The results are expressed as the increase in paw volume (ml), which was calculated by subtracting the basal volume.

### LPS-induced inflammatory model

Twenty-one mice were assigned to seven groups: the control, LPS, escin (3.6 mg/kg), LPS plus dexamethasone (4.0 mg/kg) and three LPS plus escin groups (0.9, 1.8 and 3.6 mg/kg). The corresponding drugs or saline were administered intravenously 2 h prior to the LPS treatment (40 mg/kg, intravenously). Six hours subsequent to the LPS injection, fresh liver tissues (60 mg) were homogenized in an ice-cold lysis buffer (Beyotime Institute of Biotechnology), with a 1:100 volume of phenylmethylsulfonyl fluoride (PMSF). The homogenate was centrifuged at 14,000 × g for 5 min at 4°C, and then 50 *μ*g of the proteins obtained were separated using 10% sodium dodecyl sulfate-polyacrylamide gel electrophoresis (SDS-PAGE) gels. The blots were subsequently transferred to nitrocellulose membranes, and incubated in blocking buffer (5% skimmed milk in Tris-buffered saline solution with Tween 20), prior to further incubation with rabbit polyclonal anti-NF-κB p65 in diluent buffer (both purchased from Beyotime Institute of Biotechnology) overnight at 4°C (1:1,000 dilution). Following this, the blots were incubated with the corresponding secondary antibody conjugated with horseradish peroxidase, for 2 h at room temperature.

Anti-β-actin antibody (Beyotime Institute of Biotechnology) was used as the loading control, and the blots were then developed by enhanced chemiluminescence (Amersham Biosciences, Piscataway, NJ, USA). Densitometric techniques were applied to quantify the protein band densities (ImageJ software; Rasband WS, US National Institute of Health, Bethseda, MD, USA), which were expressed as relative densitometric units of the corresponding β-actin control.

### Acetic acid-induced capillary permeability model

One hundred and twenty mice were randomly divided into four groups: the control, escin (3.6 mg/kg), diclofenac (3.6 mg/kg) and escin plus diclofenac (1.8 and 3.6 mg/kg, respectively) groups. The mice were treated with the same volume of normal saline solution or drug via the tail vein. At 10 min, and 0.5, 1, 2, 4, 6, 8, 10, 16 and 24 h subsequent to the drug administration, three mice in each group were injected intravenously with 0.2 ml 1.0% Evans Blue (Sigma, St. Louis, MO, USA) in saline solution via the tail vein, before 0.2 ml 1% (v/v) acetic acid in saline solution was injected intraperitoneally. Twenty minutes later, the mice were sacrificed, and the viscera were exposed and irrigated with 3 ml distilled water, which was then filtered through glass wool into 10 ml volumetric flasks. Each flask was made up to a 10 ml final volume with distilled water, and 0.1 ml NaOH solution (0.1 M) was added. The absorption of the final solution was measured at 590 nm using an automated enzyme-linked immunosorbent assay (ELISA) reader (Synergy™ HT Multi-Mode Microplate Reader; BioTek Instruments, Inc., Winooski, VT, USA). The inhibition level was calculated using the following formula: Inhibition (%) = (1−Ac/At) × 100, where Ac and At are the average absorptions of the saline-treated control group and the drug-treated group, respectively.

### Statistical analysis

All data are expressed as the mean ± standard error of the mean (SEM). The statistical significance of differences between the groups was determined by analysis of variance (ANOVA), followed by the Student’s t-test. P<0.05 was considered to indicate a statistically significant difference.

## Results

### Effects of escin on carrageenan-induced paw edema in rats

**Figure 1.**
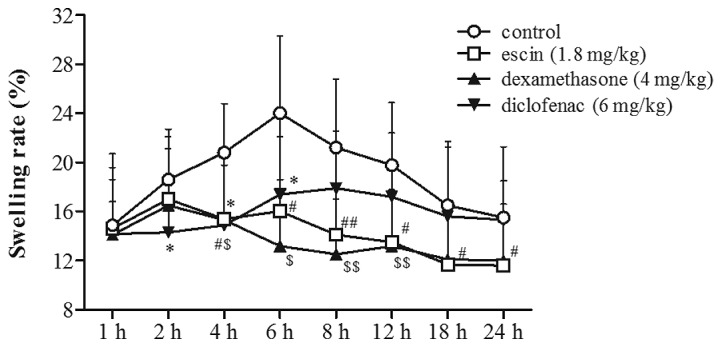
Time-effect curves of escin, dexamethasone and diclofenac in carrageenan-induced paw edema in rats. Results are expressed as the mean ± standard error of the mean. ^*^P<0.05, diclofenac compared with the control group; ^#^P<0.05 and ^##^P<0.01, escin compared with the control group; ^$^P<0.05 and ^$$^P<0.01, dexamethasone compared with the control group.

Treatment with escin (1.8 mg/kg) significantly inhibited the development of paw edema from 4 to 24 h following administration (P<0.05). Similarly, dexamethasone (4.0 mg/kg) significantly reduced the carrageenan-induced paw edema from 4 to 12 h following administration (P<0.05). In addition, the mean values of the swelling rate in the dexamethasone group at 18 and 24 h (12.1±4.7 and 12.0±4.6%, respectively) were equivalent to those of the escin group (11.7±3.9 and 11.6±3.6%, respectively). Unlike escin or dexamethasone, diclofenac (6.0 mg/kg) significantly inhibited the development of paw edema from 2 to 6 h following administration (P<0.05; Fig. 1).

### Effects of escin on NF-κB protein expression in LPS-treated mice

**Figure 2.**
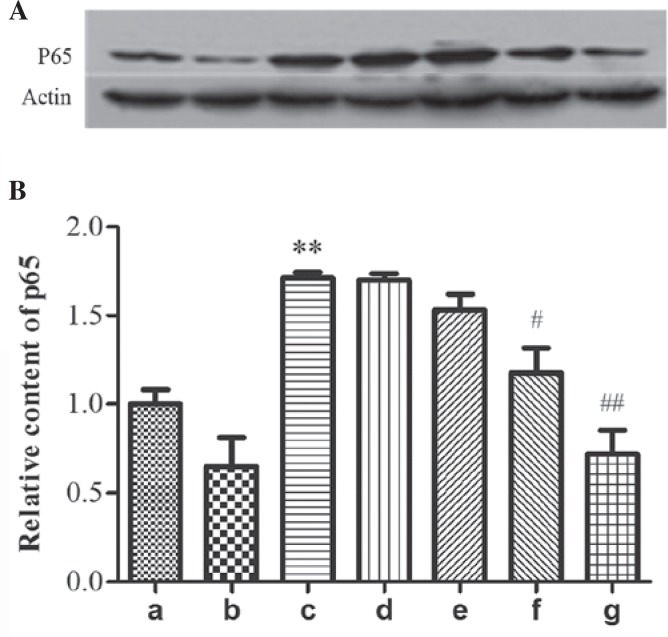
Effect of escin on the nuclear factor-κB (NF-κB) p65 protein expression level in LPS-treated mice. A) Protein expression of p65, relative to the β-actin control. B) Relative intensity of the p65 protein band, normalized to β-actin. The mice were assigned to seven groups: (a) control; (b) escin (3.6 mg/kg); (c) LPS; (d) LPS plus dexamethasone (4.0 mg/kg); and (e-g) LPS plus escin (0.9, 1.8 and 3.6 mg/kg, respectively). The results are presented as the mean ± standard error of the mean of three independent experiments. **P<0.01, compared with the control group; ^#^P<0.05 and ^##^P<0.01, compared with the LPS group.

The administration of LPS increased the protein expression level of the NF-κB p65 subunit significantly (P<0.01), unlike the administration of escin alone, which did not significantly affect the expression of the p65 subunit in the livers of the mice (P>0.05). In the LPS plus escin groups, 1.8 and 3.6 mg/kg escin significantly inhibited the protein expression of the NF-κB p65 subunit that was induced by the LPS administration (P<0.05 and P<0.01, respectively; Fig. 2).

### Anti-inflammatory effects of escin plus diclofenac on a mouse model of acetic acid-induced capillary permeability

**Figure 3.**
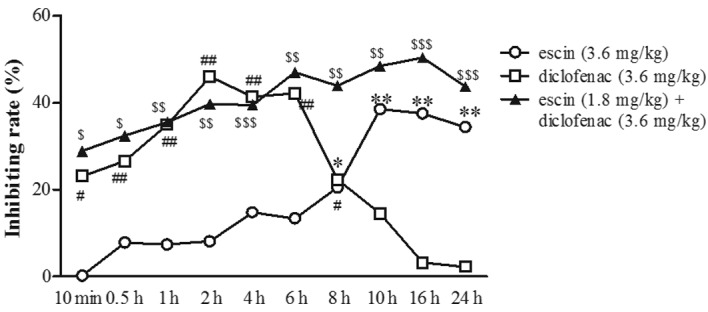
Effect of diclofenac on the anti-inflammatory activity of escin in a mouse model of acetic acid-induced capillary permeability. The results are expressed as absorption inhibiting rates, and are compared with the control group. ^*^P<0.05 and ^**^P<0.01, escin compared with the control group; ^#^P<0.05 and ^##^P<0.01, diclofenac compared with the control group; ^$^P<0.05, ^$$^P<0.01 and ^$$$^P<0.001, coadministration of escin and diclofenac compared with the control group.

Diclofenac (3.6 mg/kg) significantly inhibited the acetic acid-induced capillary permeability from 10 min to 8 h following administration. A similar effect was observed with escin (3.6 mg/kg), which significantly inhibited the capillary permeability from 8 to 24 h following administration. The coadministration of escin and diclofenac (1.8 and 3.6 mg/kg, respectively) significantly inhibited the capillary permeability from 10 min to 24 h following administration (Fig. 3).

## Discussion

The GR is a member of the nuclear receptor family that is activated by GCs. Upon activation, the GR translocates to the nucleus and inhibits proinflammatory transcription factors, such as NF-κB, thereby reducing the inflammatory reaction (17,18).

In a previous study, we demonstrated that escin elevated the expression of the GR, and reduced the liver injury induced by endotoxins in mice (4). However, it has remained unclear whether the anti-inflammatory mechanism of escin involves the GR/NF-κB signaling pathway. In the present study, we showed that escin exhibited potent GC-like anti-inflammatory effects against carrageenan-induced paw edema and acetic acid-induced capillary permeability. In addition, we demonstrated that escin significantly inhibited the expression of p65 in the livers of mice with LPS-induced sepsis. These results indicate that the anti-inflammatory effects of escin may occur through the GR/NF-κB pathway.

PGs are generated from the action of cyclooxygenases (COXs) on arachidonic acid. Diclofenac, a non-steroidal anti-inflammatory drug, potently inhibits the activity of COXs (19) and reduces the arachidonic acid level in plasma (20), and thus greatly inhibits the biosynthesis of PGs, such as PGD, PGE and PGF. An increase in PGF2α secretion from vascular endothelial cells has traditionally been considered as a critical factor for the anti-inflammatory effect of escin, and this has been documented in the description of the drug. However, in our present study, the results demonstrated that diclofenac did not exert any negative influence on the anti-inflammatory effect of escin. This indicated that the effect exerted by escin was not dependent on the increase in PGF2α secretion.

With the results from our present and previous studies, we may conclude that escin exhibits potent glucocorticoid-like anti-inflammatory effects, the mechanism of which may involve GR/NF-κB pathway; however, the effects do not depend on an increase in PGF2α secretion. These results may be useful in the further elucidation of the anti-inflammatory mechanism of escin, and to rationalize its clinical application.
